# Change Takes Time: Anxiety and Depression Following Brief Psychodynamic Counseling in University Students

**DOI:** 10.3390/healthcare14172787

**Published:** 2026-09-01

**Authors:** Costanza Franchini, Gaetano Maria Sciabica, Silvia Andreassi, Mara Morelli, Antonio Chirumbolo, Anna Maria Speranza, Alexandro Fortunato

**Affiliations:** 1 Department of Dynamic and Clinical Psychology, and Health Studies, Sapienza University of Rome, 00185 Rome, Italy; costanza.franchini@uniroma1.it (C.F.); gaetanomaria.sciabica@uniroma1.it (G.M.S.); mara.morelli@uniroma1.it (M.M.); annamaria.speranza@uniroma1.it (A.M.S.); alexandro.fortunato@uniroma1.it (A.F.); 2 Department of Psychology, Sapienza University of Rome, 00185 Rome, Italy; antonio.chirumbolo@uniroma1.it

**Keywords:** university counseling, psychodynamic counseling, university students, depression, anxiety

## Abstract

**Highlights:**

**What are the main findings?**

- Brief psychodynamic university counseling was associated with a significant reduction in depressive symptoms that was maintained three months after treatment.
- Anxiety symptoms showed a delayed improvement, with significant reductions emerging only at the three-month follow-up.

**What are the implications of the main findings?**

- Repeated longitudinal assessments can capture treatment effects that may not be evident immediately after the intervention.
- Psychodynamic counseling may represent an effective university-based intervention for supporting students experiencing anxiety and depressive symptoms during emerging adulthood.

**Abstract:**

**Background/Objectives:** University students are particularly vulnerable to anxiety and depression, yet longitudinal evidence on the effectiveness of brief psychodynamic university counseling remains limited. This study evaluated the effectiveness of a brief psychodynamic counseling intervention using a four-time-point repeated-measures design, examining changes in depressive and anxiety symptoms immediately after treatment and at a three-month follow-up. **Methods:** A total of 108 university students (*M_age_* = 22.7, *SD_Age_* = 2.67; 61% female) participated in a psychodynamic intervention consisting of four weekly sessions and a follow-up session three months later. Depressive and anxiety symptoms were assessed at four time points: after the initial consultation (T0), before treatment (T1), after the intervention (T2), and after the three-month follow-up (T3), using the Beck Depression Inventory-II and the Beck Anxiety Inventory. Longitudinal changes were examined using linear mixed-effects models controlling for age and biological sex. **Results:** Depressive symptoms remained stable during the waiting period, significantly decreased following counseling, and these improvements were maintained at follow-up. Anxiety symptoms also remained stable during the waiting period and immediately after treatment, but significantly decreased at the three-month follow-up compared with all previous assessments. Biological sex significantly predicted anxiety symptoms, with male students reporting lower anxiety than female students, whereas age was not associated with either outcome. **Conclusions:** Brief psychodynamic university counseling was associated with sustained reductions in depressive symptoms and delayed improvements in anxiety symptoms. These findings support the effectiveness of brief psychodynamic counseling and highlight the importance of longitudinal follow-up assessments to capture the temporal dynamics of psychological change.

## 1. Introduction

University students represent a particularly vulnerable population to the development of a wide range of psychopathological disorders [1,2,3]. Among these, anxiety, depression and stress-related symptoms are especially prevalent, as consistently documented by different large-scale meta-analyses [4,5].

In a meta-analysis based on 66 international studies, an average prevalence of depression of 25% and of suicidal ideation of 14% was estimated, rates nearly double those of the general population [5]. Similarly, in a meta-analysis assessing rates of psychopathological distress among students worldwide during the COVID-19 pandemic, a prevalence of anxiety of 28%, depression of 32%, and stress of 31% was reported, rates higher than those in the general population, which stood at around 28% for depression, 27% for anxiety, and 8% for stress [4,6,7].

In examining the risk factors associated with psychological distress among university students, it has been highlighted that such distress arises from the interaction of multiple individual and environmental factors [5]. Among the individual factors, a history of mental health disorders, a tendency toward negative rumination, and maladaptive coping strategies increase vulnerability to anxiety and depressive symptoms. Environmental factors, in turn, include financial difficulties, social isolation, and a history of traumatic experiences.

Beyond these factors, the difficulties experienced by university students may also be rooted in the developmental stage they are navigating: emerging adulthood [8], a complex period characterized by the need to negotiate multiple developmental challenges. Emerging adulthood, typically spanning the period between 18 and 29 years of age, represents, in fact, a unique developmental stage characterized by identity exploration, increasing autonomy, instability, and the gradual assumption of adult roles [8,9]. During this period, young people are required to negotiate multiple developmental tasks, including separation from the family of origin, the establishment of intimate relationships, and the construction of an autonomous occupational identity. When the sense of uncertainty and vulnerability inherent in this developmental period is accompanied by low self-esteem, difficulties in emotion regulation, or low perceived self-efficacy, the risk of developing internalizing disorders—such as anxiety and depression—can increase substantially [5,10].

Since for many individuals these developmental challenges coincide with university attendance, university students must simultaneously cope with normative developmental transitions and the specific demands of academic life, including performance pressure, institutional transitions, uncertainty regarding future career opportunities, financial concerns, and the need to establish new social networks [11,12]. The interaction between these developmental and contextual demands makes university students particularly vulnerable to psychological problems [5,13,14].

Poor mental health during university years has consequences extending well beyond emotional suffering. Anxiety and depressive symptoms have been associated with poorer academic performance, reduced academic self-efficacy, lower life satisfaction, impaired interpersonal functioning, diminished quality of life, and an increased risk of academic dropout [15,16,17].

In response to these growing mental health needs, universities worldwide have progressively expanded psychological support services, among which university counseling has become one of the most accessible and widely implemented interventions [13,18]. Unlike long-term psychotherapy, university counseling is specifically designed to address developmental crises and emotional difficulties through brief interventions that can be delivered to a large number of students within university settings. Rather than representing a shortened form of psychotherapy, counseling constitutes a distinct intervention aimed at promoting self-awareness and supporting adaptive developmental processes [19].

Especially in a psychodynamic framework, university counseling interventions are intended not only to reduce symptoms, but also to provide a reflective space in which students can clarify the meaning of their emotional experiences, reorganize internal representations, and reactivate developmental processes that may have become stalled during emerging adulthood [12,20]. From this perspective, symptom reduction represents only one aspect of change, as counseling seeks to promote broader developmental and psychological functioning, with previous research documenting improvements not only in anxiety, depression, and psychological distress, but also in mentalizing, interpersonal functioning, academic performance, life satisfaction, reflection and reorganization processes and time perspective [21,22,23,24,25,26,27,28].

Importantly, because these changes are conceptualized as unfolding through an ongoing process of reflection and reorganization, evaluating intervention effectiveness requires methodological designs capable of capturing not only whether change occurs, but also whether it is sustained over time. This methodological issue has been highlighted in recent reviews on the effectiveness of university counseling. Specifically, it has been observed that most effectiveness studies in the literature rely on a single pre-treatment and a single post-treatment assessment, limiting the internal validity of the available evidence [29]. Similarly, the need for longitudinal studies incorporating repeated assessments and follow-up evaluations to provide more robust evidence regarding the stability of treatment effects over time has been highlighted [22]. A recent study demonstrated that brief psychodynamic university counseling was effective in reducing depressive symptoms, showing a significant decrease in Beck Depression Inventory-II (BDI-II) scores following the intervention [30]. However, the study included only a post-treatment assessment and therefore did not examine whether these improvements were maintained over time. Building on this evidence, a longitudinal design with four assessment time points was adopted to evaluate the effectiveness of brief psychodynamic university counseling using the Clinical Outcomes in Routine Evaluation–Outcome Measure (CORE-OM) [25]. The findings showed that students’ psychological functioning remained stable during the waiting period, whereas significant improvements in psychological distress, well-being, symptoms, and functioning emerged only after the counseling intervention and were maintained at the three-month follow-up. Similarly, repeated-assessment designs have been adopted in studies evaluating cognitive-behavioral and app-based interventions for university students, with several studies including follow-up assessments and reporting significant reductions in anxiety and depressive symptoms (e.g., [31,32]). However, to the best of our knowledge, no previous study has examined the effectiveness of brief psychodynamic university counseling on anxiety and depressive symptoms using a four-time-point longitudinal design comprising two pre-intervention assessments, one post-treatment assessment, and a three-month follow-up. In light of this evidence and the gaps identified in the literature, the present study aims to extend previous research by introducing several innovative methodological features. First, the inclusion of a follow-up assessment makes it possible to examine changes not only immediately after the end of the intervention but also three months later, thereby providing evidence on the maintenance of treatment outcomes over time. Second, the study adopts a multilevel longitudinal approach that accounts for the nested structure of repeated observations within individuals, allowing changes in anxiety and depressive symptoms to be examined at the within-person level across the different phases of the counseling process. Thus, the present study contributes to the literature not only by examining whether symptoms change following brief psychodynamic counseling, but also by providing a more fine-grained assessment of when such changes emerge and whether they persist over time in a multi-counselor university setting.

### Aims and Hypotheses

The present study sought to evaluate the effectiveness of a brief psychodynamic university counseling intervention using a repeated-measures design that included a three-month follow-up, with the aim of examining both immediate symptom reduction and the maintenance of intervention outcome over time.

Based on previous evidence on the effectiveness of university counseling, we formulated two hypotheses. First, consistent with previous findings [30], we expected depressive symptoms to significantly decrease following the counseling intervention, with these improvements being maintained at the three-month follow-up. Second, we expected anxiety symptoms to similarly decrease after counseling and remain stable at the three-month follow-up.

## 2. Materials and Methods

### 2.1. Participants

The sample consisted of 108 university students (*M_age_* = 22.7, *SD_Age_* = 2.67) who accessed a university counseling center at Sapienza University of Rome and took part in a brief psychodynamic counseling intervention, consisting of four weekly sessions followed by a follow-up interview conducted three months after the conclusion of the intervention. The majority of participants were female (61%, *n* = 66). Participants’ socio-demographic characteristics are reported in Table 1.

### 2.2. Procedure

The university counseling service at Sapienza University of Rome provides a brief intervention consisting of four weekly sessions, followed by a follow-up session scheduled three months after the conclusion of the intervention. The counseling approach is psychodynamically oriented and is informed, in particular, by the Tavistock Clinic model. Rooted in the psychoanalytic tradition of the Tavistock Clinic in London, this model conceptualizes brief counseling as a time-limited intervention aimed at fostering psychological understanding and supporting individuals in addressing developmental challenges and emotional difficulties [33]. It offers a structured space for reflection in which individuals can explore the meaning of their current difficulties, a process that is particularly relevant during developmental transitions, when making sense of emotional experiences supports the integration of continuity and change in the construction of the self [12,33,34].

Counseling interventions are delivered by clinicians enrolled in the university’s postgraduate training program in psychotherapy, who are undergoing specific training in counseling. Specifically, in the present study, 34 counselors delivered the counseling intervention. Counselors received specific training in psychodynamic counseling and participated in weekly clinical supervision. While the intervention was not strictly standardized or manualized, all counselors shared the same theoretical and clinical framework, grounded in the Tavistock Clinic model.

Prior to entering the intervention, students are required to provide informed consent and complete an intake form aimed at collecting autobiographical information. This is followed by an initial consultation held at the counseling service hub, which serves to clarify the presenting concerns and to guide students toward the most appropriate service. When presenting concerns indicate the need for more specialized or intensive interventions, students are referred to appropriate mental health services. Exclusion criteria include: (a) the presence of severe psychiatric conditions (e.g., psychotic disorders or bipolar disorder), for which referral to specialized mental health services is recommended, and (b) the presence of ongoing psychotherapy.

Consistent with the design adopted in a previous study [25], the current study adopted a longitudinal within-subject design with four repeated assessments, allowing changes in psychological symptoms to be examined across the different phases of the counseling process. The study design included four assessment time points. The first assessment (T0) was administered immediately after the initial consultation; the second assessment (T1) took place just prior to the start of the counseling sessions; the third assessment (T2) was conducted upon completion of the four-session intervention; and the final assessment (T3) occurred immediately following the follow-up session, scheduled three months after the last counseling session. By including two assessments before and two after the intervention, the design allowed changes associated with counseling to be distinguished from those occurring during the waiting period. Longitudinal changes were examined using linear mixed-effects models (LMMs), accounting for the clustering of participants within counselors. The current study was approved by the Ethics Committee of Sapienza University of Rome (protocol number: 96/2023) and conducted in accordance with the Ethical Principles for Medical Research Involving Human Subjects (Declaration of Helsinki). Written informed consent was obtained from all subjects prior to participation.

### 2.3. Measures

*Sociodemographic questionnaire.* An ad hoc questionnaire developed by the counseling service was used to collect participants’ sociodemographic and academic information, including biological sex, age, access motivation, university course, degree program, and whether students were inside or outside the prescribed time for completing their degree.

*Beck Depression Inventory–II (BDI-II)* [35,36]. The BDI-II is a self-report measure developed to assess the severity of depressive symptomatology in adolescents and adults, with reference to the preceding two weeks. The instrument comprises 21 items rated on a 4-point Likert scale ranging from 0 (absence of symptoms) to 3 (severe symptomatology). Item scores, which refer to a somatic-affective dimension (e.g., loss of pleasure, fatigue, reduced energy) and to a cognitive dimension (e.g., negative self-evaluation, self-criticism, feelings of worthlessness), are summed to yield a total score ranging from 0 to 63, with higher scores indicating greater severity of depressive symptoms. Cronbach’s alpha coefficients indicated excellent internal consistency across all time points (T0 = 0.891; T1 = 0.891; T2 = 0.943; T3 = 0.951).

*Beck Anxiety Inventory (BAI)* [36,37]. The BAI is a self-report questionnaire designed to assess the severity of anxiety symptoms experienced over the previous week. Items are rated on a 4-point Likert scale, ranging from 0 (absence of symptoms) to 3 (maximum symptom severity). The instrument includes 21 items assessing the severity of anxiety symptoms, including somatic manifestations (e.g., trembling, sweating) and cognitive symptoms (e.g., fear of losing control, fear that something terrible might happen). Item scores are summed to yield a total score ranging from 0 to 63, with higher scores indicating greater severity of anxiety symptoms. In the present study, the BAI demonstrated high internal consistency across all assessment time points (T0 = 0.916; T1 = 0.916; T2 = 0.951; T3 = 0.889).

### 2.4. Statistical Analysis

All the analyses were conducted with the software Jamovi version 2.4.11 [38]. First, participants’ sociodemographic characteristics were assessed with descriptive statistics. Then, two sets of linear mixed models using the GAMLj3 module [39] were performed to assess, respectively, depression symptoms and anxiety symptoms across each time point (T0, T1, T2, T3) for each participant. These models were employed to account for the nested structure of the data, with repeated measurements nested within participants and participants nested within counselors. Age and biological sex were included as covariates in each model. When significant main effects of time were observed, Bonferroni-adjusted post hoc comparisons were conducted to examine differences between assessment time points.

## 3. Results

**Figure 1:**
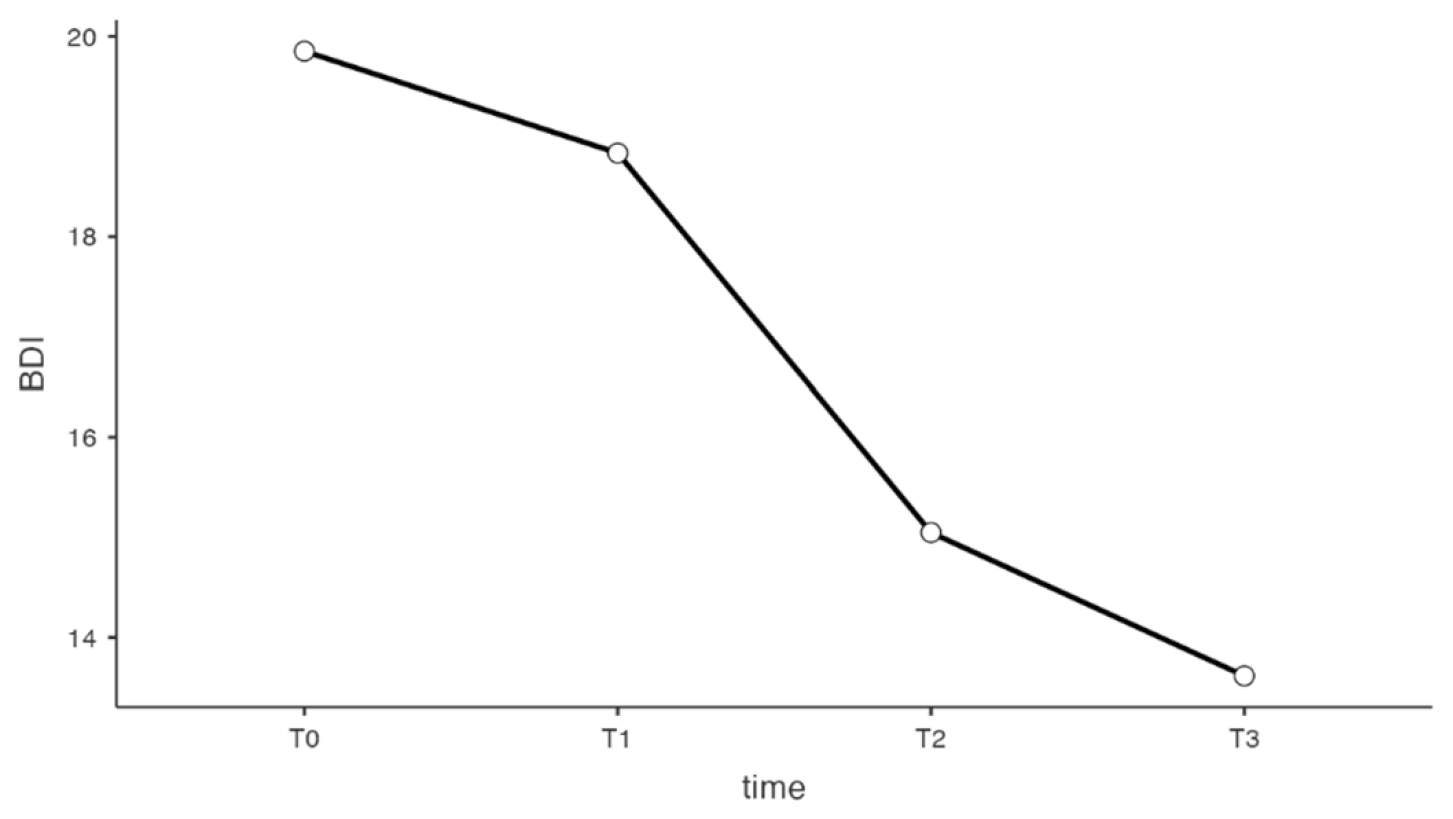
Changes in depressive symptoms over the four time points.

To examine changes in depressive symptoms over time, a linear mixed model was estimated including time (T0, T1, T2, and T3) as a fixed effect, while controlling for age and biological sex and accounting for repeated observations within participants. A significant main effect of time emerged, F(3, 319) = 16.72, *p* < 0.001, whereas neither age, F(1, 105) = 0.88, *p* = 0.350, nor biological sex, F(1, 105) = 0.002, *p* = 0.964, significantly predicted BDI-II scores. Bonferroni-adjusted post hoc comparisons indicated no significant difference between the two pre-intervention assessments (T0 and T1), suggesting that depressive symptoms remained stable during the waiting-list period. In contrast, depressive symptoms significantly decreased following the counseling intervention, with BDI-II scores significantly lower at T2 than at both T0 (*p* < 0.001) and T1 (*p* = 0.002). These improvements were maintained at the three-month follow-up, as BDI-II scores at T3 remained significantly lower than those at both T0 and T1 (both *p* < 0.001), while no significant difference emerged between T2 and T3 (*p* = 0.998), indicating that the reduction in depressive symptoms was sustained over time. See Figure 1.

The fixed effects explained 5.9% of the variance in depressive symptoms (marginal R^2^ = 0.059), whereas the full model explained 54.4% of the variance (conditional R^2^ = 0.544). The intraclass correlation coefficient (ICC) indicated that 51.5% of the variance in BDI-II scores was attributable to between-participant differences. See Table 2.

**Figure 2:**
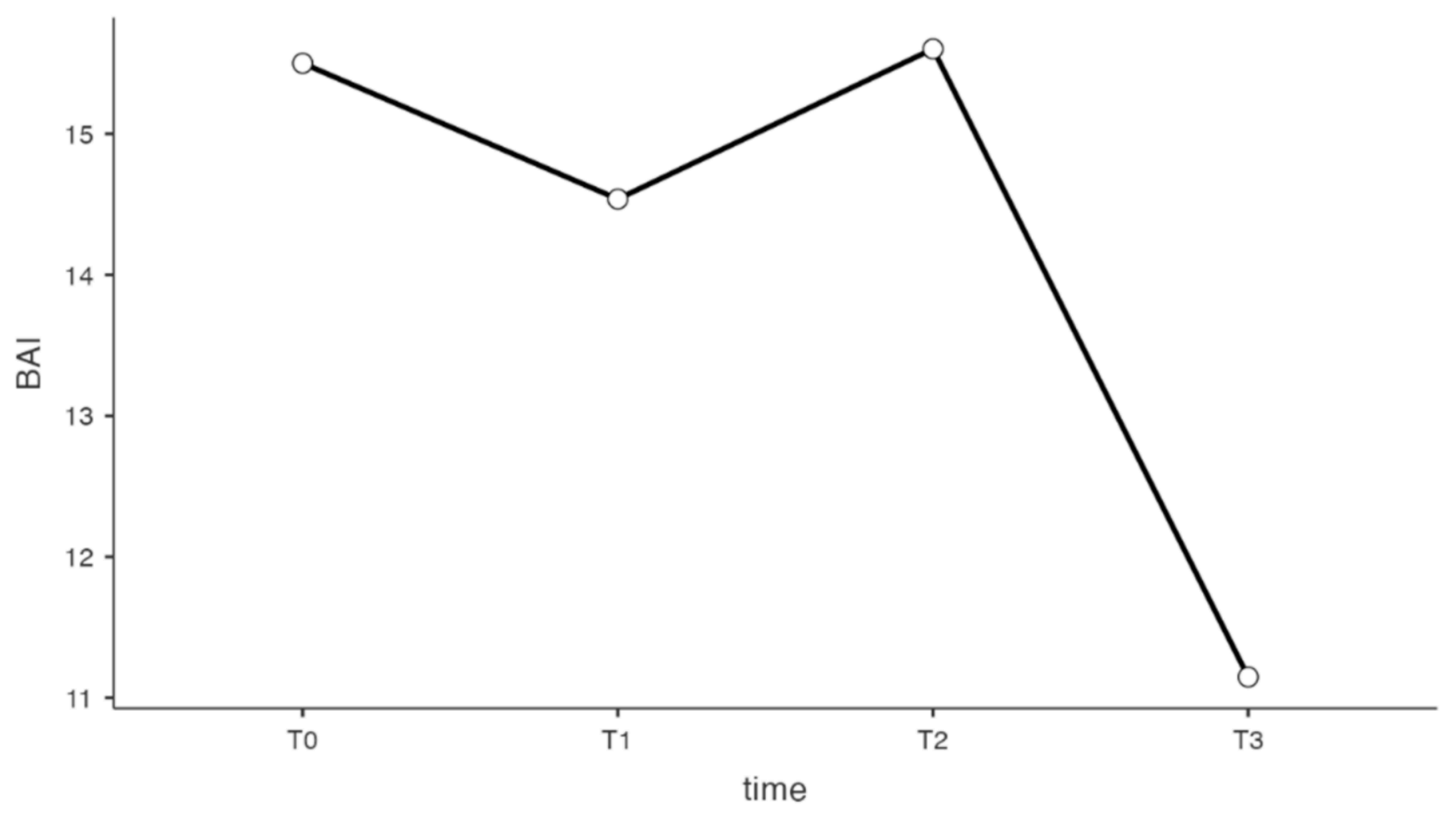
Changes in anxiety symptoms over the four time points.

A second linear mixed model was estimated to examine changes in anxiety symptoms across the four assessment points. A significant main effect of time emerged, F(3, 319) = 7.36, *p* < 0.001. Biological sex significantly predicted anxiety symptoms, F(1, 105) = 6.30, *p* = 0.014, with male students reporting lower BAI scores than female students, whereas age was not significantly associated with anxiety symptoms, F(1, 105) = 0.07, *p* = 0.790. Bonferroni-adjusted post hoc comparisons indicated no significant differences between T0 and T1, T0 and T2, or T1 and T2, suggesting that anxiety symptoms remained stable during both the waiting-list period and the counseling intervention. Significant reductions emerged only at the three-month follow-up, with BAI scores at T3 significantly lower than those at T0 (*p* < 0.001), T1 (*p* = 0.012), and T2 (*p* < 0.001). These findings indicate that, unlike depressive symptoms, improvements in anxiety became evident only after the conclusion of the counseling intervention. See Figure 2.

The fixed effects explained 5.9% of the variance in anxiety symptoms (marginal R^2^ = 0.059), whereas the full model explained 55.3% of the variance (conditional R^2^ = 0.553). The intraclass correlation coefficient (ICC) indicated that 52.5% of the variance in BAI scores was attributable to between-participant differences. See Table 3.

Because no variance was attributable to counselors (ICC = 0.000), the counselor-level random effect was omitted from the final models, and repeated observations were modeled as nested within participants only.

## 4. Discussion

The increasing prevalence of psychological distress among university students has highlighted the importance of university counseling services as a key resource for addressing students’ mental health needs [13,18]. Although a substantial body of literature has demonstrated the effectiveness of university counseling in reducing psychological symptoms [13,25,26], recent evidence has highlighted the need for longitudinal studies employing rigorous methodological designs, repeated assessments, and follow-up evaluations to establish the stability of treatment effects over time [22,29]. This need is particularly relevant for psychodynamic counseling, where change is conceptualized as a gradual process of psychological reorganization that may continue beyond the conclusion of the intervention, making repeated assessments essential for adequately evaluating its effectiveness.

The present study was designed to address this gap by examining the effectiveness of a brief psychodynamic university counseling intervention in reducing anxiety and depressive symptoms using a repeated-measures design that included two pre-intervention assessments, a post-treatment assessment, and a three-month follow-up.

Consistent with our first hypothesis, depressive symptoms significantly decreased following the counseling intervention. This finding corroborates previous evidence indicating that university counseling interventions are effective in reducing depressive symptomatology among students [13,22,25,26,30]. Importantly, this reduction remained stable at the three-month follow-up, suggesting that the benefits of the counseling intervention persisted beyond its conclusion. Previous research across different clinical contexts has similarly documented improvements in both depressive and anxiety symptoms following psychological interventions [32].

The maintenance of intervention outcomes is particularly relevant in the context of brief interventions, where an important question concerns whether improvements reflect only temporary symptom relief or more enduring psychological change. Psychodynamic theory assumes that meaningful psychological change involves modifications in the ways individuals understand, regulate, and integrate their emotional experiences [40]. Accordingly, the stability of depressive symptom improvement observed at follow-up suggests that the counseling process may have promoted enduring psychological reorganization rather than merely reducing distress during the treatment period. Furthermore, the absence of significant changes between the two pre-treatment assessments strengthens this interpretation, indicating that symptom reduction is unlikely to be attributable to spontaneous remission or the simple passage of time, but is more plausibly associated with participation in the counseling intervention itself.

From a developmental perspective, depressive symptoms during emerging adulthood may often reflect difficulties in negotiating central developmental tasks, including identity consolidation, increasing autonomy from the family of origin, the establishment of intimate relationships, and the construction of a coherent sense of self [8,9,41]. Within a psychodynamic framework, counseling does not primarily aim to address symptoms; rather, it provides a reflective relational space in which students can explore the emotional meanings underlying their distress, reorganize internal representations, and resume developmental processes that may have become temporarily stalled [12,19]. From this perspective, the observed reduction in depressive symptoms may reflect the reactivation of developmental resources and an increased capacity to understand and regulate emotional experiences, rather than symptom relief alone. However, these processes were not directly assessed in the present study and should therefore be regarded as possible theoretical interpretations rather than demonstrated mechanisms of change. Future studies incorporating measures of processes such as insight, mentalization, and emotion regulation could help determine whether these mechanisms contribute to symptom improvement following brief psychodynamic counseling.

Our second hypothesis was partially supported since the temporal pattern differed from that observed for depressive symptoms. Anxiety symptoms did not significantly decrease immediately after the counseling intervention but showed a significant reduction at the three-month follow-up. Thus, unlike depressive symptoms, improvements in anxiety appeared to emerge more gradually. Rather than simply reflecting a lack of intervention effectiveness, this pattern may point to meaningful differences in the temporal dynamics through which depressive and anxiety symptoms respond to brief psychodynamic counseling. One possible interpretation is that the counseling process initially promotes greater awareness of the emotional conflicts and developmental challenges underlying students’ presenting concerns, a process that, from a psychodynamic perspective, does not necessarily translate into immediate symptom relief. On the contrary, becoming more aware of one’s internal conflicts may initially maintain the subjective experience of anxiety while simultaneously initiating a process of psychological reorganization. The reflective work fostered during counseling may therefore require time to be integrated into everyday functioning, particularly in relation to anxiety, which is closely linked to the sense of instability and uncertainty that often characterizes emerging adulthood [8,9,12]. As students progressively integrate these new understandings and apply them to their personal experiences, reductions in anxiety may emerge more gradually, becoming evident only after the conclusion of the intervention.

This delayed improvement is compatible with the view that meaningful psychological change unfolds as a progressive process of reorganization rather than as an immediate and linear reduction in symptoms. According to Dynamical Systems Research, therapeutic interventions first promote greater flexibility and openness to new emotional and relational experiences, while symptom improvement may emerge only after these new patterns have been progressively integrated into a more adaptive psychological organization [42]. This interpretation is also consistent with psychodynamic conceptions of change, according to which the effects of treatment continue to unfold after termination through ongoing processes of reflection and integration [40].

Beyond demonstrating the effectiveness of the intervention, this temporal pattern also highlights the importance of considering psychological change as a dynamic process rather than a simple pre–post difference. As suggested by the dynamic systems perspective, meaningful change is better understood through its trajectory over time, since symptom improvement may emerge only after a process of progressive reorganization rather than as a linear reduction immediately following treatment [43]. These findings therefore underscore the importance of longitudinal follow-up assessments, as clinically meaningful improvements may not be fully observable immediately after the intervention has concluded.

With regard to anxiety symptoms, an additional finding of interest concerns the significant effect of biological sex, with male students reporting significantly lower levels of anxiety than female students. This pattern is consistent with a substantial body of literature showing that women are more likely to experience and report anxiety symptoms than men, both in the general population and among university students [1,5].

An additional finding of particular interest concerns the absence of variability in intervention outcomes attributable to individual counselors. Despite the intervention being delivered by 34 different clinicians, the random-effects analyses indicated no meaningful counselor-level variance in changes in either depressive or anxiety symptoms. This finding suggests that the effectiveness of the intervention was largely independent of the specific counselor providing it. One possible explanation is that the standardized training and regular clinical supervision characterizing the counseling service may have promoted a consistent implementation of the intervention across clinicians, as previously reported [25].

From a clinical perspective, the present findings further support the implementation of brief psychodynamic counseling services within university settings as an effective intervention for students experiencing anxiety and depressive symptoms. At the same time, they underscore the importance of evaluating counseling outcomes through rigorous longitudinal designs that include repeated assessments and follow-up evaluations. More broadly, these findings support the view that university counseling should not be considered merely a brief symptom-focused intervention, but rather a developmental intervention capable of fostering enduring psychological resources that help students navigate the complex emotional challenges characteristic of emerging adulthood.

## 5. Limitations and Future Developments

Despite the innovative aspects of the present study, some limitations should be acknowledged. First, psychological symptoms were assessed exclusively through self-report questionnaires, which may be affected by social desirability. Future studies could integrate clinician-rated assessments (e.g., clinical interviews) or observer-based measures to provide a more comprehensive evaluation of treatment-related changes.

Second, participants were recruited from a single university counseling service and represented students seeking help within a psychodynamically oriented intervention. Moreover, individuals presenting with severe psychiatric disorders or already engaged in psychotherapy were referred to other mental health services. These characteristics may limit the generalizability of the findings to other university contexts, counseling models, or clinical populations. Future studies should therefore seek to replicate these findings across different institutions and cultural contexts.

Third, the absence of a control group represents an important methodological limitation of the present study. Although the inclusion of two pre-intervention assessments allowed us to examine symptom stability during the waiting period, the present design does not allow causal conclusions regarding the effectiveness of the intervention. Future research should employ randomized controlled trials and comparative designs to more rigorously evaluate the effectiveness of brief psychodynamic counseling and to determine whether the observed changes are specifically attributable to the intervention. Moreover, longer follow-up periods are needed to examine the stability of these changes over time and to strengthen the generalizability of the present findings.

Fourth, this methodological limitation is particularly relevant to the interpretation of the delayed reduction in anxiety symptoms observed at follow-up. Although anxiety symptoms remained stable across the two pre-intervention assessments and immediately after counseling, it cannot be excluded that their subsequent reduction may partly reflect natural fluctuations or recovery over time, repeated measurement effects, changes in students’ personal or academic circumstances, or other experiences occurring during the three-month follow-up period. Future controlled studies are therefore needed to clarify the extent to which this delayed improvement can be attributed to the counseling intervention and to better understand the processes underlying its temporal trajectory.

Finally, although the present study demonstrated significant improvements in anxiety and depressive symptoms, it did not directly examine the mechanisms underlying these changes. Future research should therefore investigate the processes through which brief psychodynamic counseling promotes symptom improvement, with particular attention to the mechanisms that may account for the distinct temporal trajectories observed for anxiety and depressive symptoms.

## 6. Conclusions

This study examined the effectiveness of a brief psychodynamic counseling intervention in reducing depressive and anxiety symptoms among 108 university students, across four assessment points, including a three-month follow-up. The results show two distinct temporal trajectories. Depressive symptoms remained stable during the waiting period, decreased significantly after the intervention, and this reduction was maintained at follow-up. Anxiety symptoms, on the other hand, showed a delayed reduction, becoming significant only at the three-month follow-up, highlighting a different timing of change. An association also emerged between biological sex and anxiety levels, with lower levels observed among male students, while age was not associated with the outcomes considered. Overall, the reduction in symptoms and the different timing of change constitute significant findings: improvement in depressive symptoms emerged after the intervention and remained stable, while improvement in anxiety symptoms became evident only during the subsequent period. These results provide a positive indication of symptom improvement and underscore the importance of longitudinal assessments, as changes may emerge at different times depending on the symptom considered. Future studies should also examine psychological processes such as insight, mentalization, and emotion regulation to clarify their possible contribution to symptom improvement.

## Figures and Tables

**Table 1:** Descriptive statistics.

	Total Sample (*n* = 108)%
Biological Sex	61% F (66)
Mean age	22.67 (±2.67)
Access motivation	
Emotional problems	59.3% (64)
University-related problems	32.4% (35)
Other	8.3% (9)
University courses	
Architecture	2.8% (3)
Economics	5.6% (6)
Pharmacy and medicine	5.6% (6)
Law	4.6% (5)
Civil and industrial engineering	10.2% (11)
Information engineering, computer science and statistics	8.3% (9)
Literature and philosophy	14.8% (16)
Medicine and dentistry	9.3% (10)
Medicine and psychology	6.5% (7)
Mathematical, physical and natural sciences	25.9% (28)
Political sciences, sociology and communication	6.5% (7)
Degree program	
Bachelor’s degree	62.0% (67)
Master’s degree	32.4% (35)
PhD course	5.6% (6)
Inside prescribed time	80.6% (87)

Biological sex was coded as: 0 = F, 1 = M.

**Table 2:** Linear mixed model: change in BDI-II total score over time.

				Confidence Intervals				
Names	Effect	Estimate	SE	Lower	Upper	*df*	t	*p*
Fixed coefficients								
Intercept		16.836	0.854	15.157	18.515	105	19.709	<0.001
T1	T1–T0	−1.018	1.026	−3.036	0.999	319	0.992	0.322
T2	T2–T0	−4.805	1.026	−6.823	−2.788	319	−4.682	**<0.001**
T3	T3–T0	−6.237	1.032	−8.267	−4.208	320	−6.041	**<0.001**
Age		0.299	0.319	−0.328	0.927	105	0.938	0.350
Biological sex	1–0	0.079	1.739	−3.338	3.497	105	0.045	0.964
Random Components					Variance	SD	ICC	
Students’ intercept					60.5	7.78	0.515	
Residual					56.9	7.54		
Model Fit					R^2^	*df*	LRT X^2^	*p*
Conditional					0.544	6	156.689	<0.001
Marginal					0.059	5	47.998	<0.001
Post-Hoc Comparison: Time				Difference	SE	*df*	t	*p*
T0 vs. T1				1.02	1.03	0.992	319	1.000
T0 vs. T2				4.81	1.03	4.682	319	<0.001
T0 vs. T3				6.24	1.03	6.042	320	<0.001
T1 vs. T2				3.79	1.03	3.690	319	0.002
T1 vs. T3				5.22	1.03	5.055	320	<0.001
T2 vs. T3				1.43	1.03	1.387	320	0.998

Note. Biological sex was coded as follows: 0 = female; 1 = male; SE = standard error; *df* = degrees of freedom; SD = standard deviation; ICC = intraclass correlation coefficient. Significant values are in bold.

**Table 3:** Linear mixed model: change in BAI total score over time.

				Confidence Intervals				
Names	Effect	Estimate	SE	Lower	Upper	*df*	t	*p*
Fixed coefficients								
Intercept		14.196	0.913	12.402	15.991	105	15.549	<0.001
T1	T1–T0	−0.963	1.080	−3.085	1.159	319	−0.891	0.373
T2	T2–T0	0.101	1.080	−2.020	2.224	319	0.094	0.925
T3	T3–T0	−4.353	1.086	−6.488	−2.218	320	−4.008	**<0.001**
Age		0.090	0.341	−0.579	0.761	105	0.266	0.790
Biological sex	1–0	−4.663	1.858	−8.316	−1.011	105	−2.509	**0.014**
Random Components					Variance	SD	ICC	
Students’ intercept					69.6	8.34	0.525	
Residual					62.9	7.93		
Model Fit					R^2^	*df*	LRT X^2^	*p*
Conditional					0.553	6	161.721	<0.001
Marginal					0.059	5	27.954	<0.001
Post-Hoc Comparison: Time				Difference	SE	*df*	t	*p*
T0 vs. T1				0.963	1.08	0.8919	319	1.000
T0 vs. T2				−0.102	1.08	−0.0943	319	1.000
T0 vs. T3				4.353	1.09	4.0083	320	**<0.001**
T1 vs. T2				−1.065	1.08	−0.9863	319	1.000
T1 vs. T3				3.390	1.09	3.1216	320	**0.012**
T2 vs. T3				4.455	1.09	4.1021	320	**<0.001**

Note. Biological sex was coded as follows: 0 = female; 1 = male; SE = standard error; *df* = degrees of freedom; SD = standard deviation; ICC = intraclass correlation coefficient. Significant values are in bold.

## Data Availability

The data presented in this study are available on request from the corresponding author. The data are not publicly available due to privacy reasons.
